# Identification of pigmented *Serratia marcescens* symbiotically associated with *Rhynchophorus ferrugineus* Olivier (Coleoptera: Curculionidae)

**DOI:** 10.1002/mbo3.377

**Published:** 2016-06-01

**Authors:** Maria Scrascia, Carlo Pazzani, Franco Valentini, Marta Oliva, Valentina Russo, Pietro D'Addabbo, Francesco Porcelli

**Affiliations:** ^1^ Department of BiologyUniversity of Bari “Aldo Moro”Via E. Orobona 470125BariItaly; ^2^ Mediterranean Agronomic Institute of Bari/International Centre for Advanced Mediterranean Agronomic StudiesVia Ceglie 970010Valenzano (BA)Italy; ^3^ Department of Soil Sciences, of Plants and FoodUniversity of Bari “Aldo Moro”Via G. Amendola165/A 70126BariItaly

**Keywords:** 16S rDNA, antimicrobial activity, bacterial red pigment, housekeeping genes, *Rhynchophorus ferrugineus*, *Serratia marcescens*

## Abstract

To characterize red pigment‐producing bacteria (RPPB) regularly released during oviposition by red palm weevil (RPW), RPPB were recovered from eggs deposited in apples supplied as substrate for oviposition. The presence of RPPB was also detected from gut, the reproductive apparatus of dissected adult and virgin insects and from pupal cases collected within infested palms. RPPB were also identified all along the tissue of these palms. Analysis of the 16S rDNA,* gyrB*,* rpoB*,* recA*, and *groEL* sequences assigned RPPB to the species *Serratia marcescens*. RPPB exhibited an antimicrobial activity assessed by the agar well diffusion method against a number of gram‐positive and gram‐negative bacteria. In this study, we first report the identification of a red pigment‐producing *S*. *marcescens* as extracellular symbiont of RPW. Route of transmission, detection within different organs, and a wide spread along the infested palm tissue, suggested *S. marcescens* is present as extracellular symbiont in different developmental stages of the RPW. Additionally, the antimicrobial activity exhibited versus *Bacillus* spp., *Paenibacillus* spp., and *Lysinibacillus* spp., reported as insect pathogens and potential candidates for biocontrol agents, could ascribe for *S. marcescens* a potential protective role.

## Introduction

*Rhynchophorus ferrugineus* Olivier (Coleoptera: Curculionidae), also known as red palm weevil (RPW), is considered one of the most invasive pests of major cultivated palms. RPW originated from Sundaland zoogeographical regions and nowadays, it is widely distributed in Oceania, Asia, Africa, and Europe (EPPO bulletin 2008). In the Mediterranean basin, it was first detected in the early 1990s and since then, it has been spreading progressively, invading most of the Mediterranean countries (Al‐Eryan et al. 2010). Its main hosts are coconut (*Cocos nucifera* L.), Canary island date palm (*Phoenix canariensis* hort. ex Chabaud), date palm (*Phoenix dactylifera* L.), and oil palm (*Elaeis guineensis* Jacq) (Cox 1993; Giblin‐Davis et al. 2013). The economic importance of RPW is based on the host plant shifting from wild palms to alternative cultivated hosts and/or on the RPW ability to infest a considerable number of palm species at a very low population density (Porcelli et al. 2012).

The RPW biological cycle involves distinct development stages where symbiotic microorganisms play an important role (Tagliavia et al. 2014). For instance, culturable bacteria associated both with larval gut and wet fermenting frass have been suggested to play a beneficial role for RPW (e.g., plant polymers breakdown within the larva digestive system) (Butera et al. 2012). Studies on bacteria associated with RPW and the potential role played by these microorganisms have been widely recommended. This might undoubtedly contribute to supply further knowledge on the biological cycle of this insect and also to the development of new strategies for RPW control.

In our laboratory, during campaigns for RPW tree‐injection control in Italy, Malta, Morocco, Spain, and Syria (Porcelli et al. 2009, 2013), red pigment‐producing bacteria (RPPB) associated with egg chambers were regularly observed in a number of experiments where apples were supplied to RPWs as substrate for oviposition. The systematic detection of RPPB on eggs for these bacteria suggested a vertical transmission by egg smearing during oviposition. This route of transmission (identified for extracellular symbionts) has been commonly reported for various insect orders, including Coleoptera, Diptera, Hymenoptera, and Hemiptera (Salem et al. 2015). Additionally, the production of pigments is of particular biological interest. In fact, pigments are bacterial secondary metabolites with many antagonistic effects, for example, antimicrobial, anticancer, and immunosuppressive (Chang et al. 2011; Vaishnav and Demain 2011; Petersen and Tisa 2013). Secondary metabolites with antimicrobial activity have been detected in vertically transmitted extracellular symbionts which protect their insect hosts against pathogens or predators (Brownlie and Johnson 2009; Seipke et al. 2012).

We than focused our study on the characterization of RPPB released during RPW oviposition and their location within RPW. An antibacterial activity against a number of gram‐positive and gram‐negative bacteria was also established.

## Methods

### Insect collection and bacterial isolation from apple egg chambers

**Figure 1:**
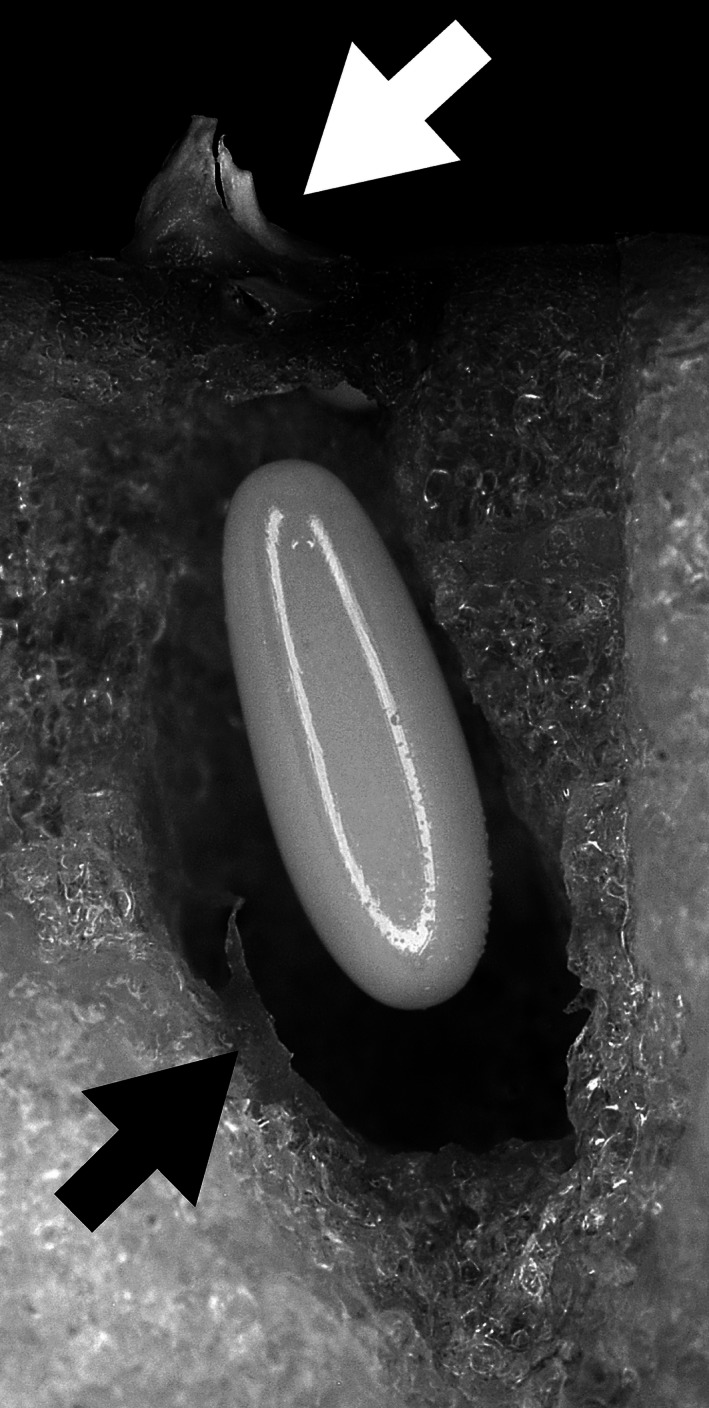
Longitudinal parasagittal section of a *Rhynchophorus ferrugineus* egg chamber. The white arrow shows the top plug on the apple surface. The black one shows the brownish matter on the chamber wall. An egg is into the chamber itself. The white line on the egg is the ring‐flash reflection.

Five healthy and active RPW females were collected from Bari (Puglia) by pheromone and fruit lured traps (ferrugineol + banana) in 2009 (three adults) and 2014 (two adults). Females were placed in single breeding boxes with one apple as food and oviposition substrate. Each female drilled several chambers in the apple, laying one egg per chamber. Each egg was laid into an egg chamber purposely dig underneath the apple peel by RPW female (Fig. 1). Each chamber was stopped by a small white plug that could be clearly spotted on the apple surface (Fig. 1: white arrow). Five egg chambers per female were tested cutting out an apple chunk around each egg‐laying pit with a sterile scalpel. Each chunk was split and sterile cotton sticks were used to swab material from the egg surface and from the chamber (Fig. 1: black arrow). The collected materials were streaked onto sterile Nutrient Agar (NA, CM0003 OXOID, Milan, Italy) and incubated at 28–30°C under aerobic conditions for 24–48 h in a growth cabinet. Uninfested apple peel and pulp from five apples were plated as control. RPPB were purified by streaking single red colonies onto fresh NA and maintained at −80°C in nutrient broth (NB) containing 15% glycerol.

### Insect collection, RPW dissection, and bacterial isolation

Eleven RPW females were field collected from 2013 to 2014: in 2013, five adults were caught from Salerno (Campania) urban area by pheromone and fruit lured traps (ferrugineol + banana); in 2014, six insects were caught from a felled RPW‐infested Canary Palm in Bari (Puglia).

In 2015, infested palm tissues, pupal cases, and adults were collected from two felled RPW‐infested Canary Palms in South Italy: ten pupal cases with five samples of infested palm tissue from Castrovillari (Calabria); fourteen pupal cases with five samples of infested palm tissue and 15 adults (six males and nine females) from Bari (Puglia).

All adults were kept on *P. canariensis* chips and dissected within 5 days from collection. The pupal cases were individually kept in single boxes until use.

Insects were anesthetized with CO2 before dissection. The surface was sterilized by 5% sodium hypochlorite/water solution and sterility was checked by streaking the cuticle of sterilized insects on NA. The entire reproductive apparatus and/or gut were extracted from the abdomen. Gut and selected regions of female (ovarioles, calyces, sperm receptacle, copulatory pouch, and ovipositor) and male (testes, aedeago, and ducts) reproductive apparatus were cut with sterile scalpels, individually rinsed three times in sterilized water, and laid on NA plates. Plates were incubated at 28–30°C under aerobic conditions for 24–48 h in a growth cabinet.

RPPB were purified by streaking single red colonies onto fresh NA and then preserved at −80°C in NB containing 15% glycerol.

### Molecular analysis

**Figure 2:**
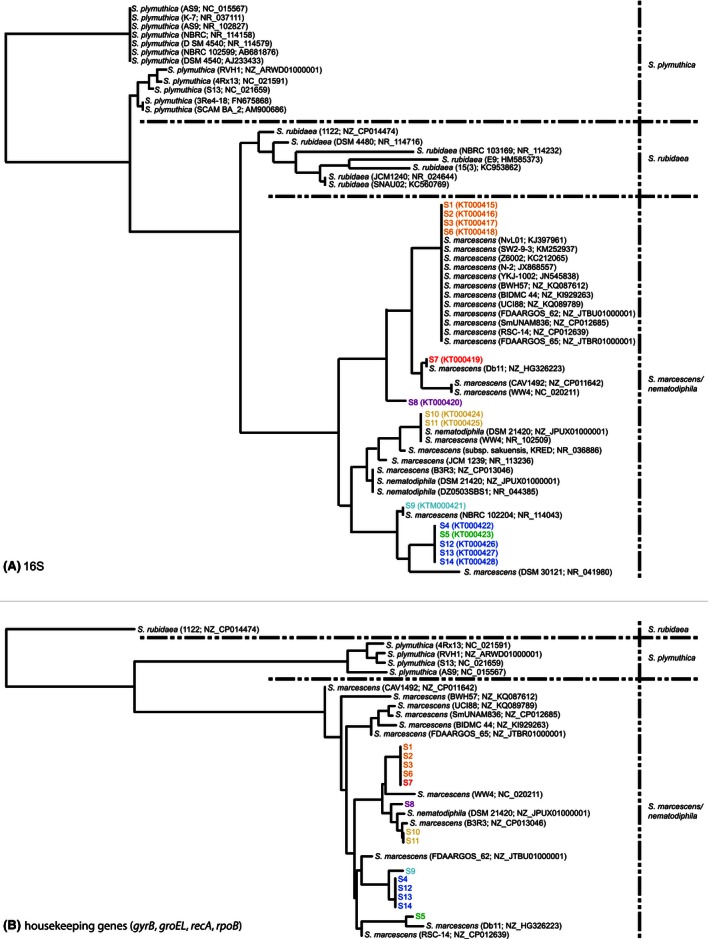
Phylogenetic trees based on the neighbor‐joining. (A) relationship inferred from the alignment of 973 bp of 16S rDNA. (B) relationship inferred from the alignment of 3285 bp of the *gyrB* (808 bp), *rpoB* (830 bp), *recA* (823 bp), and *groEL* (824 bp). *S. nematodiphila* is a red pigment‐producing species, recently reported, and phylogenetically strictly related to the species *marcescens* (Zhang et al. 2009).

Genomic DNA was extracted from cultured bacteria by the cetyltrimethylammonium bromide method (Murray and Thompson 1980) and used as template for PCR which was performed as previously reported (Valle et al. 2005). The universal primers P1 (Muyzer et al. 1993) and R1378 (Heuer et al. 1997) were used to amplify the V3–V8 hypervariable regions of the bacterial 16S rDNA gene (Yu and Morrison 2004). Sequencing of the amplified fragments was performed by Beckman Coulter Genomics (France). The 16S rDNA sequences were searched for nucleotide identity in the Ribosomal Database Project collection (RDP; Release 11, Update 2, March 2014) by Sequence Match (http://rdp.cme.msu.edu/seqmatch) (Cole et al. 2003) and in the GenBank database by NCBI Web BLAST (http://blast.ncbi.nlm.nih.gov/Blast.cgi). Phylogenetic relationship among the 16S rDNA gene sequences of both the 14 RPW isolates and a selection of GenBank records belonging to red pigment‐producing *Serratia* species *marcescens/nematodiphila*,* rubidaea*, and *plymuthica* (see Fig. 2 for accession numbers) were computed by neighbor‐joining method as implemented in the Tree Viewoption of the NCBI Web Blast (http://www.ncbi.nlm.nih.gov/blast/treeview/treeView.cgi). Near‐complete sequences of the 16S rDNA gene and sequences of the *recA, rpoB, groEL*, and *gyrB* housekeeping genes were extracted from the entire genomes of the four isolates S1, S5, S8, S13 (unpublished data) sequenced by next‐generation sequencing using Illumina HiSeq platform. Primers specific for *recA, rpoB, groEL*, and *gyrB* were designated in this study and reported in Table S8. PCRs were performed in a total volume of 50 *μ*L containing 50–100 ng of total DNA, 1X PCR buffer (20 mmol/L Tris‐HCl, 50 mmol/L KCl, 1.5 mmol/L MgCl_2_; pH8.4), 125 *μ*mol/L of each deoxynucleoside triphosphate (dNTP), 20 *μ*mol/L of each primer, and 1 U *Taq* polymerase Platinum (Life Technologies, Milan, Italy). Cycling conditions were: 94°C for 5 min; 30 cycles of 94°C for 30 sec, 60°C for 1 min and 72°C for 1 min; final extension at 72°C for 5 min.

The 16S rDNA, *recA, rpoB, groEL*, and *gyrB* sequences produced in this study and their annotations are available at GenBank database under the accession numbers listed in Table 1 and in Tables S1–S6.

**Table 1:** Bacterial isolates from the red palm weevil (RPW) female reproductive apparatus

Isolate	Year	Place of isolation	RPW compartment	16S rDNA accession number
S1	2009	Bari	Egg chamber	KT000415
S2	2009	Bari	Egg chamber	KT000416
S3	2009	Bari	Egg chamber	KT000417
S4	2014	Bari	Egg chamber	KT000422
S5	2014	Bari	Egg chamber	KT000423
S6	2013	Salerno	Copulatory pouch	KT000418
S7	2013	Salerno	Ovipositor	KT000419
S8	2013	Salerno	Ovipositor	KT000420
S9	2013	Salerno	Copulatory pouch	KT000421
S10	2014	Bari	Copulatory pouch	KT000424
S11	2014	Bari	Copulatory pouch	KT000425
S12	2014	Bari	Copulatory pouch	KT000426
S13	2014	Bari	Copulatory pouch	KT000427
S14	2014	Bari	Sperm receptacle	KT000428

### Antimicrobial susceptibility and biochemical tests

The ability to inhibit bacterial growth was assessed using the agar well diffusion method (Lertcanawanichakul and Sawangnop 2008) with some modifications. Five milliliters of bacterial red‐pigmented cultures were obtained after inoculation in NB and incubation for 4–5 days at 28–30°C. Bacteria to be challenged were prepared as described in Clinical and Laboratory Standards Institute (CLSI) protocol VET01‐A4 (CLSI 2013). A sterile cotton swab was dipped into the liquid culture for 2 sec (to allow for saturation), pressed against the side of the tube to remove excess liquid, and then streaked onto the surface of NA. Wells 8–10 mm in diameter were punched in the agar with a sterile tip and 20 *μ*L of each red culture was directly filled into the wells. The plates were incubated at 28–30°C for 18–24 h and the diameter of the inhibition zone was measured in millimeters. Assays were performed in triplicate and sterile water was used as negative control.

The antibacterial activity of the RPPB was assessed against five diverse species of gram‐negative strains and five diverse species of gram‐positive strains: *Escherichia coli* strains LE392 and ATCC25922, *Vibrio cholerae* strain El Tor 787, *Bacillus megaterium* strain ATCC14581, *Bacillus pumilus* strain ATCC7061, and *Staphylococcus aureus* strain ATCC25923 were from our laboratory collection. Two clinical isolates of *Salmonella typhimurium*, two clinical isolates of *Klebsiella pneumoniae*, and one clinical isolate of *Acinetobacter baumannii* were provided by the U.O.C. Microbiologia e Virologia Azienda Ospedaliero‐Universitaria Consorziale Policlinico di Bari (Italy); *Lysinibacillus* spp. and *Paenibacillus* spp. isolates were recently isolated in our laboratory from environmental stone samples and identified by analysis of the16S rDNA partial sequence.

Biochemical tests were performed using the API 20E (Biomérieux, Rome, Italy) according to manufacture instructions. The ability of the isolates to produce the enzyme catalase was assessed by placing 1 drop of 3% hydrogen peroxidase (H_2_O_2_) on a microscope slide containing a small amount of fresh bacterial culture. The immediate bubble formation indicated a positive result. All biochemical tests were performed in triplicate.

## Results

The presence of RPPB, transmitted by egg smearing and never detected from uninfested apple peels and pulps, was confirmed from each analyzed RPW egg chamber. One red pigment‐producing isolate was randomly selected from each female used in this experiment (named from S1 to S5) (Table 1). Analysis of the 16S rDNA sequences assigned the selected five RPPB to the genus *Serratia* (family *Enterobacteriaceae*), with the highest similarity found for the species *marcescens* (Tables S1 and S2). We have then explored the presence of RPPB within the RPW female reproductive apparatus. Eleven adult females were dissected and RPPB were found from the reproductive apparatus of nine of them. RPPB were never found on control plates where the insect surface cuticle was streaked before dissection. From each female positive for RPPB, one isolate was selected (named from S6 to S14) and analysis of the 16S rDNA sequences also assigned these strains to the genus *Serratia* with the highest similarity found for the species *marcescens* (Tables S1 and S2). Identification of the genus *Serratia* was also confirmed by the API20E system: all isolates were tested and they exhibited biochemical properties consistent with the genus *Serratia* (Table S7).

The genus *Serratia* includes, at least, 10 species (Grimont and Grimont 2006). We further characterized all RPPB at the species level by analysis of the *groEL*,* gyrB*,* recA*, and *rpoB* housekeeping gene sequences. BLAST analysis (http://blast.ncbi.nlm.nih.gov/Blast.cgi) of most of the housekeeping gene sequences, as well as that suggested by the 16S rDNA sequences, classified our isolates into the species *marcescens* (Tables S3–S6). The phylogenetic trees based on the 16S rDNA and the concatenated housekeeping gene sequences arranged our strains within the *S*. *marcescens* cluster. This cluster was clearly distinct from those of the other known red pigment‐producing *Serratia* species (namely *plymuthica* and *rubidaea*) (Grimont and Grimont 2006; de Araujo et al. 2010) (Fig. 2).

*S. marcescens* has also been reported, at least for some red pigment‐producing strains, to exhibit an antimicrobial activity against some gram‐positive and gram‐negative bacteria (Ibrahim et al. 2014; Lapenda et al. 2015). We then verified if this was also the case for our isolates. A clear inhibition zone on bacterial growth was observed around all the *Serratia* isolates when challenged with gram‐positive bacteria (*Bacillus* spp., *S. aureus, Paenibacillus* spp., and *Lysinibacillus* spp.) (Table 2). A variable activity was detected among different gram‐negative bacteria: *A. baumannii, and V. cholerae* were resistant; *E. coli*,* S. typhimurium*, and *K. pneumoniae* showed a variable phenotype with respect to the *S. marcescens* strain, were challenged to (Table 2). Our data confirmed the antimicrobial activity exhibited versus *Bacillus* spp and *S. aureus* strains described by previous studies (Ibrahim et al. 2014; Lapenda et al. 2015). Additionally, our study further extended this spectrum of susceptibility versus isolates of the *Paenibacillus* and *Lysinibacillus* genera. With regard to gram‐negative bacteria, our data were informative versus pathogenic isolates of *V. cholerae, S. typhimurium*, and *K. pneumoniae* species. Interestingly, the different susceptibility phenotype showed by *E. coli, S. typhimurium*, and *K. pneumoniae,* could imply different molecules with antimicrobial activity produced by some RPPB.

**Table 2:** Antimicrobial activity of RPPB isolated from red palm weevil females^a^

Challenged bacteria (No of strains)	S1; S3; S7	S2; S4; S5; S6; S8; S9; S10; S11; S12; S13; S14
Gram negative		
* A. baumannii* (1)	0	0
* S. typhimurium* (2)	8–10	0
* K. pneumoniae* (2)	9–11	0
* V. cholerae* (1)	0	0
* E. coli* (2)	10–14	0
Gram positive		
* B. megaterium* (1)	13–16	12–20
* B. pumilus* (1)	14–15	13–24
* S. aureus* (1)	10–12	13–17
* Lysinibacillus* spp. (1)	12–14	9–16
* Paenibacillus* spp. (1)	12–17	15–20

a The diameter of inhibition zone on microbial growth is reported in millimeters.

Detection of RPPB was further extended to the reproductive apparatus of adults RPW males, gut of males and females, virgin males and females (picked up from pupal cases), inner part of pupal cases, and infested palm tissues (Table 3).

**Table 3:** Presence of RPPB within infested palm tissues and red palm weevils collected in South Italy in 2015

Source (Number)			N of positive
Palm	Female	Male	
Pupal cases (8)			8
Infested palm tissue (10)			10
	Reproductive apparatus from virgin (5)		1
	Reproductive apparatus from adult (6)		5
	Gut from virgin (6)		0
	Gut from adult (8)		7
		Reproductive apparatus from virgin (7)	3
		Reproductive apparatus from adult (6)	2
		Gut from virgin (5)	0
		Gut from adult (5)	3

In infested palm tissues and pupal cases, RPPB was always identified while, from the reproductive apparatus and gut, the isolation of RPPB varied. In adults insects, RPPB was detected with an higher frequency in females than in males. Conversely, in virgin insects, RPPB was only detected from the reproductive apparatus of males and very seldom in that of females.

## Discussion

The long‐term coexistence of insect and bacteria has involved the development of nutritional and defense benefits between the partners, which have had profound and ecological consequences (Salem et al. 2015). The persistence and evolution of such partnerships has necessarily implied efficient transfer mechanisms of bacteria across host generations whether directly (vertical mode), indirectly (horizontal mode), or through their combination (mixed mode) (Ebert 2013). Among the vertical mode of transmission, smearing bacteria over the surface of newly deposited eggs is one of the most commonly reported routes for the transfer of extracellular symbionts. This has been widely reported for the orders of Coleoptera, Diptera, Hymenoptera, and Hemiptera (Salem et al. 2015). In particular, in the order Coleoptera, bacteria transmission of symbionts has been proposed to occur from males to the bursa copulatrix of females, and subsequently to offspring via eggs smearing (Steinhaus 1946; Iverson et al. 1984).

In this study, we first report the identification of a red pigment‐producing extracellular symbiont (*S. marcescens*) detected in both gut and reproductive apparatus of RPW and regularly released during oviposition. *S. marcescens* as extracellular symbiont, has been very rarely reported in insects. In this role, to the best of our knowledge, it was only identified in Sugar Beet Root Maggot, *Tetanops myopaeformis* (van Röder), where its transfer was reported to occur from the male to the female reproductive apparatus and from the latter to offspring via internal infiltration of egg chorion and external smearing of eggs during oviposition (Iverson et al. 1984).

Detection of the genus *Serratia* in RPW has recently been reported (Montagna et al. 2015). However, the aim of this study was to investigate the effects of diet on the microbiota of RPW and, a part for its detection in RPW and the absence in uninfested palm tissue, no further data were stated for this genus. Our findings of *S*. *marcescens* released during oviposition and its detection on infested palm tissue, pupal cases, and virgin insects, could propose this aerobic heterotrophic and facultative anaerobic bacterium as extracellular symbiont present in different developmental stages of the RPW. Additionally, the diffuse presence of *S*. *marcescens* within the infested palms highlighted the ability of this bacterium to replicate and spread along the palm tissue.

RPW is a solitary insect with no or limited contact between adult and developing individuals. Solitary insects, additionally to their own defenses, can make use of symbionts to better protect themselves, offspring, or nutritional resources against pathogens, predators, parasites, or parasitoids (Kellner 2002; Kaltenpoth et al. 2005; Brownlie and Johnson 2009). This protection can be mediated by different mechanisms which include the production of antimicrobials. A clear example has been reported for the digger wasp, *Philathus triangulum* (“beewolves” Hymenoptera, Crabronidae) and its symbiont ‘*Candidatus* Streptomyces philanthi’ (Koehler et al. 2013). The antimicrobials, produced by this symbiont, allow a long‐term protection of wasp's offspring against pathogens. It is noteworthy that the symbiont transmission to offspring also occurred during oviposition.

The red‐pigmented *S. marcescens* strains identified in RPW exhibited antimicrobial activity against a number of both gram‐positive and gram‐negative bacteria. Of particular relevance was the inhibition growth detected versus *Bacillus* spp., *Paenibacillus* spp. and *Lysinibacillus* spp. which have been reported as insect pathogens and potential candidates for biocontrol agents (McSpadden Gardener 2004; Berry 2012; Lacey et al. 2015).

The interactions between microorganisms and hosts have always been the object of intensive studies. In particular, studies on the mutualistic relationships between bacteria and insects have progressively revealed the relevant role played by the formers on the life cycle of their hosts. The identification of red pigment‐producing *S. marcescens* as extracellular symbiont of RPW shall contribute to the knowledge on a mutualistic relationship between bacteria and RPW. At present time, the role played by this extracellular symbiont still has to be established, although beneficial functions of symbiont (as the one protective against potential pathogens) seem to be consistent with our findings.

## Conflict of Interest

The authors declare that they have no conflict of interests.

## Supporting information


**Tables S1.** Blast of 16S rDNA sequences versus NCBI 16S‐specific database.
**Tables S2.** Blast of 16S rDNA sequences versus NCBI Genomes database.
**Table S3.** Blast of groEL sequences versus NCBI Genomes database.
**Table S4.** Blast of gyrB sequences versus NCBI Genomes database.
**Table S5.** Blast of recA sequences versus NCBI Genomes database.
**Table S6.** Blast of rpoB sequences versus NCBI Genomes database.

**Table S7.** Biochemical results of the API 20E test and catalase assay: + indicates positive result; − indicates negative result.
**Table S8.** Primers designated in this study.
