# Supplementary material for: Identification of pigmented Serratia marcescens symbiotically associated with Rhynchophorus ferrugineus Olivier (Coleoptera: Curculionidae)
Source: Microbiologyopen. 2016 Jun 1;5(5):883–90. doi: 10.1002/mbo3.377 (PMC5061723; doi:10.1002/mbo3.377)
Supplement: Supplementary file 2 — Table S7. Biochemical results of the API 20E test and catalase assay: + indicates positive result; − indicates negative result. Table S8. Primers designated in this study. [file MBO3-5-883-s002.docx]

**Table S7**: biochemical results of the API 20E test and catalase assay

| **Test** | **S1; S2; S3; S6; S10; S11; S12; S13** | **S4; S5; S9; S14** | **S7** | **S8** |
| --- | --- | --- | --- | --- |
| Beta-galactosidase (ONPG) | + | + | + | + |
| Arginine dihydrolase (ADH) | - | - | - | - |
| Lysine decarboxylase (LDC) | + | + | + | + |
| Ornithine decarboxylase (ODC) | + | + | + | + |
| Citrate utilization (CIT) | + | + | + | + |
| H2S production (H2S) | - | - | - | - |
| Urease (URE) | - | - | - | - |
| Tryptophane deaminase (TDA) | - | - | - | - |
| Indole production (IND) | - | - | - | - |
| Acetoin production (VP) | + | + | + | + |
| Gelatinase (GEL) | + | + | - | + |
| Glucose fermentation (GLU) | + | + | + | + |
| Mannitol fermentation (MAN) | + | + | + | + |
| Inositol fermentation (INO) | + | - | + | - |
| Sorbitol fermentation (SOR) | + | + | + | + |
| Rhamnose fermentation (RHA) | - | - | - | - |
| Sucrose fermentation (SAC) | + | + | + | + |
| Melibiose fermentation (MEL) | + | + | - | - |
| Amygdalin fermentation (AMY) | + | + | + | + |
| Arabinose fermentation (ARA) | + | + | + | - |
| Cytochrome-oxidase (OX) | - | - | - | - |
| Catalase | + | + | + | + |

**Table S8**: primers used in this study

| **groEL** |  |  |  |  |  |  |  |  |  |
| --- | --- | --- | --- | --- | --- | --- | --- | --- | --- |
| **Primer pair** | **Sequence (5'->3')** | **Template strand** | **Length** | **Start** | **Stop** | **Tm** | **GC%** | **Self complementarity** | **Self 3' complementarity** |
| Forward primer | CCGCAACGTAGTGCTGGATA | Plus | 20 | 104 | 123 | 60.18 | 55.00 | 4.00 | 2.00 |
| Reverse primer | CCAGCTCCAGACCGATCTCT | Minus | 20 | 927 | 908 | 60.75 | 60.00 | 6.00 | 2.00 |
| **Product length** | 824 |  |  |  |  |  |  |  |  |
|  |  |  |  |  |  |  |  |  |  |
| **gyrB** |  |  |  |  |  |  |  |  |  |
| **Primer pair** | **Sequence (5'->3')** | **Template strand** | **Length** | **Start** | **Stop** | **Tm** | **GC%** | **Self complementarity** | **Self 3' complementarity** |
| Forward primer | TGGTATTCGAGGTTGTGGAC | Plus | 20 | 116 | 135 | 57.25 | 50.00 | 4.00 | 2.00 |
| Reverse primer | ACCTTGGCCTTCTTGCTGTA | Minus | 20 | 923 | 904 | 59.23 | 50.00 | 4.00 | 2.00 |
| **Product length** | 808 |  |  |  |  |  |  |  |  |
|  |  |  |  |  |  |  |  |  |  |
| **recA** |  |  |  |  |  |  |  |  |  |
| **Primer pair** | **Sequence (5'->3')** | **Template strand** | **Length** | **Start** | **Stop** | **Tm** | **GC%** | **Self complementarity** | **Self 3' complementarity** |
| Forward primer | CTCCATGGACGTGGAAACGA | Plus | 20 | 102 | 121 | 60.04 | 55.00 | 8.00 | 0.00 |
| Reverse primer | GTTACAGGCATTCGCCTTGC | Minus | 20 | 924 | 905 | 60.18 | 55.00 | 5.00 | 2.00 |
| **Product length** | 823 |  |  |  |  |  |  |  |  |
|  |  |  |  |  |  |  |  |  |  |
| **rpoB** |  |  |  |  |  |  |  |  |  |
| **Primer pair** | **Sequence (5'->3')** | **Template strand** | **Length** | **Start** | **Stop** | **Tm** | **GC%** | **Self complementarity** | **Self 3' complementarity** |
| Forward primer | TCGTTCCAGAAGTTTATCGAGCA | Plus | 23 | 100 | 122 | 60.06 | 43.48 | 4.00 | 2.00 |
| Reverse primer | ATCAGTTCGCCGGTATTGGT | Minus | 20 | 929 | 910 | 59.46 | 50.00 | 4.00 | 1.00 |
| **Product length** | 830 |  |  |  |  |  |  |  |  |
